# New Insights into the Genome Organization of Yeast Double-Stranded RNA LBC Viruses

**DOI:** 10.3390/microorganisms10010173

**Published:** 2022-01-14

**Authors:** Manuel Ramírez, Alberto Martínez, Felipe Molina

**Affiliations:** 1 Departamento de Ciencias Biomédicas (Área de Microbiología), Facultad de Ciencias, Universidad de Extremadura, 06006 Badajoz, Spain; amartinetp@alumnos.unex.es; 2 Departamento de Bioquímica, Biología Molecular y Genética (Área de Genética), Facultad de Ciencias, Universidad de Extremadura, 06006 Badajoz, Spain; fmolina@unex.es

**Keywords:** *Torulaspora*, killer, LBC virus, dsRNA genome, high-throughput sequencing (HTS), frameshifting nucleotide insertions or deletions (indels), xenolog

## Abstract

The yeasts *Torulaspora delbrueckii* (Td) and *Saccharomyces cerevisiae* (Sc) may show a killer phenotype that is encoded in dsRNA M viruses (V-M), which require the helper activity of another dsRNA virus (V-LA or V-LBC) for replication. Recently, two TdV-LBCbarr genomes, which share sequence identity with ScV-LBC counterparts, were characterized by high-throughput sequencing (HTS). They also share some similar characteristics with Sc-LA viruses. This may explain why TdV-LBCbarr has helper capability to maintain M viruses, whereas ScV-LBC does not. We here analyze two stretches with low sequence identity (LIS I and LIS II) that were found in TdV-LBCbarr Gag-Pol proteins when comparing with the homologous regions of ScV-LBC. These stretches may result from successive nucleotide insertions or deletions (indels) that allow compensatory frameshift events required to maintain specific functions of the RNA-polymerase, while modifying other functions such as the ability to bind V-M (+)RNA for packaging. The presence of an additional frameshifting site in LIS I may ensure the synthesis of a certain amount of RNA-polymerase until the new compensatory indel appears. Additional 5′- and 3′-extra sequences were found beyond V-LBC canonical genomes. Most extra sequences showed high identity to some stretches of the canonical genomes and can form stem-loop structures. Further, the 3′-extra sequence of two ScV-LBC genomes contains rRNA stretches. The origin and possible functions of these extra sequences are here discussed.

## 1. Introduction

Killer yeasts produce protein toxins that are lethal to sensitive yeasts. The synthesis and secretion of killer toxins by *Torulaspora delbrueckii* (Td) and *Saccharomyces cerevisiae* (Sc) requires the presence of at least two cytoplasmic dsRNA viruses that are members of the family *Totiviridae*. One is a satellite virus with a medium-size genome (V-M) that encodes the toxin, and the other is a helper virus with a large-size genome (V-LA) that provides the capsid and polymerase required for maintenance and replication of both viruses. Additionally, the role of ScV-M1 dsRNA in the maintenance of ScV-LA1 by a yet-unknown mechanism has been recently suggested [1].

A specific LA virus may support different types of satellite M viruses but usually only one type in each killer yeast strain [2,3]. LBC viruses are another type of large-size dsRNA virus that may coexist with V-LA and V-M in the cytoplasm of *S. cerevisiae*; although no helper activity is known for V-LBC in this yeast species [4,5,6,7,8]. However, a new LBC virus has recently been found in *T. delbrueckii* (TdV-LBCbarr2) that may act as a helper for two M viruses in the same yeast strain: TdV-Mbarr1 and ScV-M1 [9]. These viruses are inherited in the cytoplasm from mother yeast to daughter bud and transferred horizontally between different yeasts by mating or heterokaryon formation [10]. However, the Sc-M1 virus has been recently found in Td, which suggests that it may have been transferred horizontally between different yeasts by conventional viral infection [9].

V-LA and V-LBC genomes encode the coat protein of the virion (Gag), and an RNA polymerase is required for virus propagation which is a fusion protein (Gag-Pol) translated by a −1 ribosomal frameshifting mechanism. The slippery site for ribosomal frameshifting is “GGGUUUA” in V-LA, and “GGAUUUU” in V-LBC [8,9,11,12,13]. Several cis stem-loops located in the 3′-terminal region of LA (+)RNA are involved in its packaging and replication [3,8,9,11,12,13,14,15]. The 3′-region of V-M (+)RNA (non-coding region) contains stem-loops similar to the signals required for packaging and replication in L viruses. This allows V-M to share the replication machinery with V-L for viral propagation. Moreover, the transcription initiation signal located at the 3′-end of canonical V-L (−)RNA (3′CTTTTT in V-LA and 3′CTTAAA in V-LBC; corresponding to 5′GAAAAA and 5′GAATTT in the (+)RNA strand, respectively), is also mimicked in the (−)RNA 3′-end of V-M canonical sequence [4,6,9,14,16,17,18,19].

All V-M genomes contain a central poly(A) that divides the genome into two parts: a toxin-coding 5′-region, and a non-coding 3′-region that contains the stem-loops that mimic the signals of V-L required for packaging and replication [4,6,17]. The canonical sequences of V-M genomes share a similar organization, but no overall sequence identity. However, there are some repeated short sequences located in the non-coding 3′-region of several V-M genomes indicating a common phylogenetic origin. Interestingly, high local identity has been found among the protein sequences of some killer toxins and some unclassified proteins of several organisms, entailing a possible ancient input of RNA sequences from hosts into the toxin’s gene during the emergence of M viruses. No homologies have been found among the extra sequences located beyond the ends of the canonical sequence of several V-M genomes. However, the 3′-extra sequence of some V-M genomes contained stretches with high identity to some rRNA sequences; and the 5′-extra sequence of several viruses contained stretches almost identical to Sc-LBC2 virus or to some genomic sequence of *Vitis vinifera*, *Saccharomycopsis fibuligera*, and *Cucumis melo*. These findings evidence that M-virus RNA could recombine with cellular RNA, or RNA present in the medium (grape or melon juice), and eventually keep part of these RNA sequences attached to the ends of the viral genome to originate new virus isotypes [4,6].

*Saccharomyces* LA viruses also exhibit additional 5′ and 3′ sequences beyond the canonical genome. In some cases, these extra ssRNA stretches can form stem-loops, whose unpaired nucleotides could form intramolecular kissing complexes. It has been suggested that these extra sequences may form complex RNA structures preserving the virus ssRNA from degradation and facilitating the dsRNA synthesis [14]. Amino-acid Gag-Pol sequences of LA viruses share great identity (87–99%). Despite this, a 42-amino-acid variable sequence stretch (42–58% identity) has been described, which is located between Gag and Pol domains. This low identity stretch (LIS) does not seem to be of much importance for virus replication, and its function could simply be the separation of Gag and Pol domains in the Gag-Pol fusion protein [14,15].

New V-LBC genomes from Sc and Td yeasts have recently been sequenced using HTS techniques and subsequently characterized. Identity among the new Sc-LBC viruses was above 95% for amino acid sequences, similar to that found for previously known Sc-LBC viruses [5]. According to the percentage of identity of Gag-Pol sequence, LBC viruses were grouped in two clusters: *S. cerevisiae* cluster (95–100%) grouping all Sc-LBC viruses, and *T. delbrueckii* cluster (98.3%) grouping the two known Td-LBC viruses. Viruses from different clusters share rather a low identity rate (about 44%), despite that most viruses were isolated from the same geographical region [9]. The canonical length of TdV-LBCbarr1 and TdV-LBCbarr2 genomes is slightly shorter than that of Sc-LBC viruses (4565 vs. 4615 bp). Although these two types of virus share a rather low identity rate, their genome organization is similar [9], and also similar to that described for LA viruses [13,14]. Thus, their first ORF encodes the coat (Gag) protein of the virion, while the second ORF encodes an RNA-dependent RNA polymerase (RdRp) [5,8,9,13,14]. TdV-LBCbarr genomes also contain some important regions present in ScV-LA [5,7,13,14,15,18]: (i) a stem-loop for frameshifting located downstream from the slippery site; (ii) a stem-loop for (+)ssRNA packaging located downstream from RdRp domain; and (iii) a stem-loop for RNA replication located upstream from 3′ end [9]. These motifs are found in equivalent positions with respect to the ScV-LA genomes. Similar stem-loops seem to be also present in ScV-LBC genomes but located in different positions with respect to ScV-LA genomes. The similarity of these motifs and features of LBCbarr viruses and LA viruses could explain the helper capability of TdV-LBCbarr2 to maintain M viruses [9]. The genomes of Td-LBCbarr viruses also contain the 5′ AU-rich region present in L and M viruses. This motif seems to facilitate the “melting” of the template (−)RNA strand and the access of the RNA polymerase for conservative transcription [4,6,19,20]. This motif is 5′GAAATT in TdV-LBCbarr, which is similar to that of ScV-LBC (5′GAATTT), and different from that of ScV-LA (5′GAAAAA) [9,14]. Although both TdV-LBCbarr Gag-Pol amino acid sequences showed modest global identity with that of Sc-LBC viruses (about 44%), most of the motifs required for Gag and Pol functions in Sc-LBC viruses were also found in LBCbarr viruses [9].

This study deeply analyzes the genome sequence of LBC viruses to determine the presence of: (i) low identity stretches located in the viral canonical genomes, (ii) sequence stretches with relevant identity to genomic sequence that might have been horizontally transferred from cellular organisms (xenologs), (iii) 5′- and 3′-extra sequences, and (iv) possible interactions between these extra sequences and proximal canonical sequences to form stem-loops and intramolecular kissing complexes. All these features may help to elucidate the phylogenetic origin of these viruses.

## 2. Materials and Methods

### 2.1. Yeast Strains and Culture Media

The yeasts used in this study are shown in Table 1. The killer phenotype and presence of L and M genomes (dsRNA) in these yeast strains were previously analyzed [4,14,17]. Standard culture media were used for yeast growth [21].

### 2.2. Purification of V-LBC dsRNA from Killer Yeasts

Nucleic acid samples from killer yeasts were obtained as previously described [14,22]. The dsRNA from each yeast culture was obtained by CF-11 cellulose chromatography [23]. L and M dsRNA were separated by gel electrophoresis in 1% agarose. The 4.6 kb bands were cut from the gel and purified using RNaid^®^ Kit (MP Biomedicals, LLC, Illkirch, France).

### 2.3. cDNA Library Preparation and DNA Sequencing

The preparation of cDNA libraries and HTS (high-throughput sequencing) were performed at Unidad de Genómica Cantoblanco (Fundación Parque Científico de Madrid, Spain) as described elsewhere [14]. Random primers dTVN and dABN (Isogen Life Science, De Meern, The Netherlands) and SuperScriptIII retrotranscriptase (Thermo Fisher Scientific, Waltham, MA, USA) were used for cDNA first-strand synthesis. Subsequently, the synthesis of the second cDNA strand, end repair, adenylation in 3′-end, and TruSeq adaptors’ ligation was performed (Illumina, San Diego, CA, USA). The adaptor oligonucleotides contained signals for DNA amplification and sequencing, as well as short sequences (indices) for multiplexing in the sequencing run. Each library was amplified using a PCR enrichment procedure, ensuring that all cDNA molecules of the library contained the adaptors at both ends. The resulting libraries were denatured prior to seeding on a flow cell for sequencing on a MiSeq system by using 2 × 80–2 × 150 sequencing runs.

### 2.4. Assembly of Virus Genome Sequences

The cDNA sequences were assembled by the company Biotechvana (Technological Park of Valencia, Spain) as described elsewhere [14]. A de novo assembly was done using SOAP deNOVO2 method [24] and two Illumina libraries for each virus. Multiple assembly attempts were tried with scaffolding and an insert size of 200. The most effective Kmer value was 47. Contigs shorter than 300 nt were removed from the config file. The selected contigs were used as input to the NR database of the NCBI using the BLASTX program [25] implemented in GPRO 1.1 software [26]. High identity was found between several contigs/scaffolds and some previously known viral RNA sequences (such as V-LA and LBC dsRNA) or host transcripts. Contaminating sequences that resulted in non-homologous to known V-LBC genomes were filtered. Each virus genome was sequenced at least three times. Different samples and dates were used for each virus. Full coverage of the canonical sequence of each virus was obtained at least twice. 100% identity was obtained for all sequences from the same killer yeast strain. Viral genomes comparisons were done using only the full coverage sequences.

### 2.5. Sequence Analysis Tools

The sequence identity among nucleotide sequences of L genomes was obtained by using ClustalW(2.1) program [27], and MUSCLE(3.8) software for amino-acid sequence comparison [28]. Global alignment for identification of low identity stretches (below 50% identity in windows of 50 nucleotides or 20 amino acids in length) was done using Clone Manager 7.11 (Sci Ed Software LLC, Westminster, CO, USA), Scoring matrix: Linear (Mismatch 2, OpenGap 4, ExtGap 1 for cDNA; and BLOSUM 62 for protein). BLAST software and a data bank of nucleic acids were used to search for identities between viral genomes. Only BLAST hits with identity above 80% and length above 30 nucleotides were considered as xenologs. The MFOLD program (http://unafold.rna.albany.edu/?q=mfold/RNA-Folding-Form, (accessed on 21 December 2021)) was used to predict the folding of ssRNA [29]. The parameters used were: 37 °C as folding temperature; ionic conditions of 1M NaCl and no divalent ions; 5 as percent suboptimality number; 50 as upper bound on the number of computed foldings; 30 as maximum interior/bulge loop size; 30 as maximum asymmetry of an interior/bulge loop; and no limit for maximum distance between paired bases.

### 2.6. Nucleotide Sequences

ScV-LBC1-original [8], ScV-LBC2-S3920 [5], ScV-LA1-original [13], ScV-LA2-8F13 [5], SpV-LA28 [30] were previously analyzed by traditional techniques of cloning and sequencing. TdV-LAbarr1-EX1180, ScV-LA1-EX231, ScV-LAlus4-EX229, ScV-LAlus1-EX436, ScV-LAlusA-EX1160, ScV-LA2-EX1125, TdV-LBCbarr1-EX1180, TdV-LBCbarr2-EX1257, LBClus4-EX229, LBC1-EX231, LBC2-EX1125, and LBClusA-EX1160 have been sequenced by HTS techniques [9,14]. Table 2 shows the GenBank accession numbers of the viral genomic sequences analyzed in this study.

## 3. Results

### 3.1. Comparison of Nucleotide Canonical Sequences from TdV-LBC and ScV-LBC Genomes

**Figure 1:**
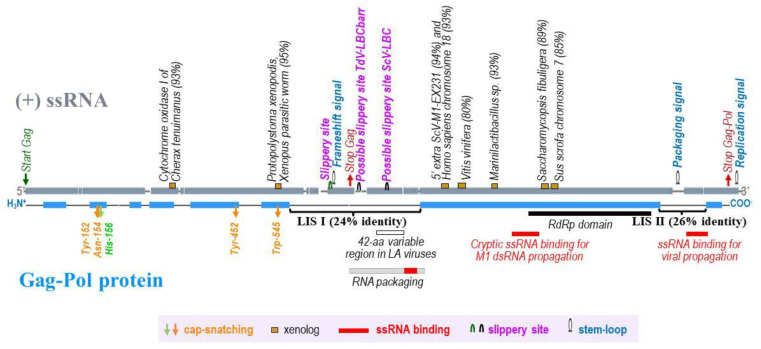
A schematic representation of TdV-LBCbarr (ssRNA and Gag-Pol) showing the two stretches of low sequence identity with Gag-Pol of ScV-LBC1-original: stretch I (LIS I) and stretch II (LIS II). Grey (ssRNA) and blue (Gag-Pol protein) shaded regions are areas of significant identity (above 50% in windows of 50 nucleotides or 20 amino acids in length) between TdV-LBCbarr2 and ScV-LBC1-original genomes. Global alignment was done using Clone Manager 7.11. Scoring matrix: Linear (Mismatch 2, OpenGap 4, ExtGap 1 for cDNA; and BLOSUM 62 for protein). Relevant RNA codons and motifs of the viral genome are displayed above the (+)ssRNA, as well as stretches (≥30 nucleotides) showing high local identity (≥80%) with xenolog sequences of cellular organisms (percentage of identity is in parenthesis). Relevant amino acids for cap-snatching and RdRp domain are shown below Gag-Pol, as well as the location of 42-amino acid variable region, ssRNA binding regions, and RNA packaging region previously described in yeast LA viruses.

In addition to the conserved slippery site found in all known LBC viruses upstream from the Gag ORF stop codon, TdV-LBCbarr genomes have a second putative in-frame translation re-initiation codon (“2151GGGGAG**ATG**A2160”) located downstream from Gag-ORF and upstream from Pol domain. An identical second putative in-frame start codon is also present in all ScV-LBC genomes, but in a different location (“2324GGGGAG**ATG**A2333”). These ATG in-frame codons are preceded by a possible slippery site, “GGGGAG”, in all cases (Figure 1 and Figure S1).

The identity between the highly-conserved RdRp-domain sequences of *T. delbrueckii* and *S. cerevisiae* LBC viruses (64–66%) was greater than that found for full Gag-Pol sequences (44%), and much greater than that found for Gag sequences (37%). In most cases, the percentage of identity between the different LBC viruses was higher when comparing the amino acid sequences of Gag-Pol than when comparing the nucleotide sequence of the genomes. The opposite occurred when comparing the sequences of TdV-LBC and ScV-LBC, which showed 44% and 64–66% identity for amino acid Gag-Pol and canonical nucleotide sequences, respectively. This exception was mainly explained by the presence of two stretches in TdV-LBCbarr Gag-Pol that show very low identity with the homologous region from Sc-LBC viruses: LIS I, from amino acid A558 to K828, and LIS II, from S1340 to I1443, which showed 24% and 26% identity with ScV-LBC1-original, respectively. Interestingly, the two ssRNA-binding domains of ScV-LA1-original Gag-Pol, that are necessary for viral propagation [31], are located in the homologous sequences of these two LIS. Additionally, the stem-loop for packaging and a region with low RNA-sequence identity are also located inside LIS II. Similar low identity stretches were not found when comparing TdV-LA and ScV-LA Gag-Pol sequences [14]. However, the 42-aa variable region, that separates Gag and Pol domains Sc-LA viruses [14,15], is fully coincident with part of TdV-LBCbarr LIS I (Figure 1).

High local identity (80–95% for stretches longer than 30 nt) was found between the nucleotide sequences of TdV-LBCbarr genomes and some xenolog mitochondrial or genomic sequences of organisms such as *Cherax tenuimanus* (93% identity, 30 nt in mitochondrial cytochrome oxidase I gene), *Xenopus* parasitic worm *Protopolystoma xenopodis* (95%, 37 nt in contig 0228346), *Homo sapiens* (93%, 30 nt in chromosome 18), *Vitis vinifera* (80%, 54 nt in contig VV78X249912.5), *Marinilactibacillus* sp. 15R (93%, 30 nt in sequence CP017761.1), *Saccharomycopsis fibuligera* (89%, 37 nt in chromosome B6), and *Sus scrofa* (85%, 40 nt in chromosome 7). Most of these putative xenolog stretches are located upstream from the highly conserved RdRp domain, except the last two that are located in this domain. High local identity was also found between a stretch of ScV-LBC2-EX1125 (94%, 66 nt) and the 5′-extra sequence previously found in ScV-M1-EX231 [4], the only case among Sc-LBC viruses. All of these stretches were found along the LBC genome but none was coincident with any of the two LIS regions (Supplementary Figure S1 and Figure 1).

### 3.2. Analysis of 5′- and 3′-Extra Sequences of TdV-LBCbarr and ScV-LBC Genomes

**Figure 2:**
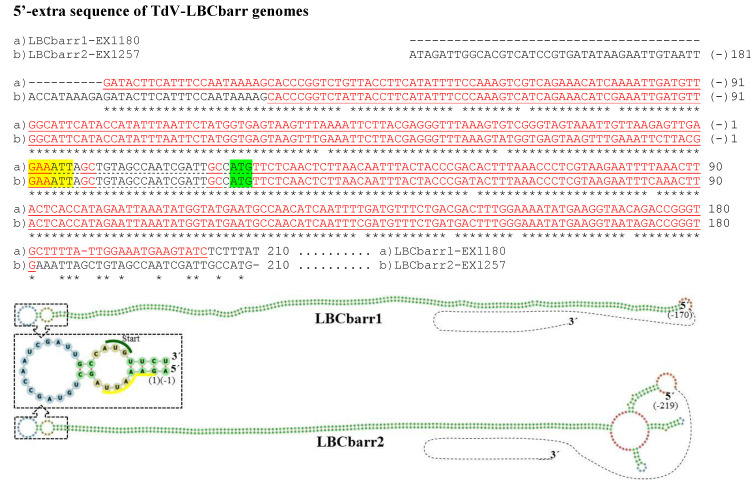
cDNA of 5′-extra sequences and proximal canonical sequences of TdV-LBCbarr1 genome from EX1180 strain and TdV-LBCbarr2 genome from EX1257 strain. The 5′-GAAATT end of the canonical sequence is yellow highlighted. The protein synthesis initiation codon of Gag-Pol is green shaded. Nucleotides of palindromic sequences are shown in red letters. The stem-loop sequences are underlined. The unpaired nucleotides of each loop are dot underlined. Asterisks (*) indicate identical nucleotides. The secondary RNA structures of possible 5′ stem-loops are shown at the bottom of the sequences.

**Figure 3:**
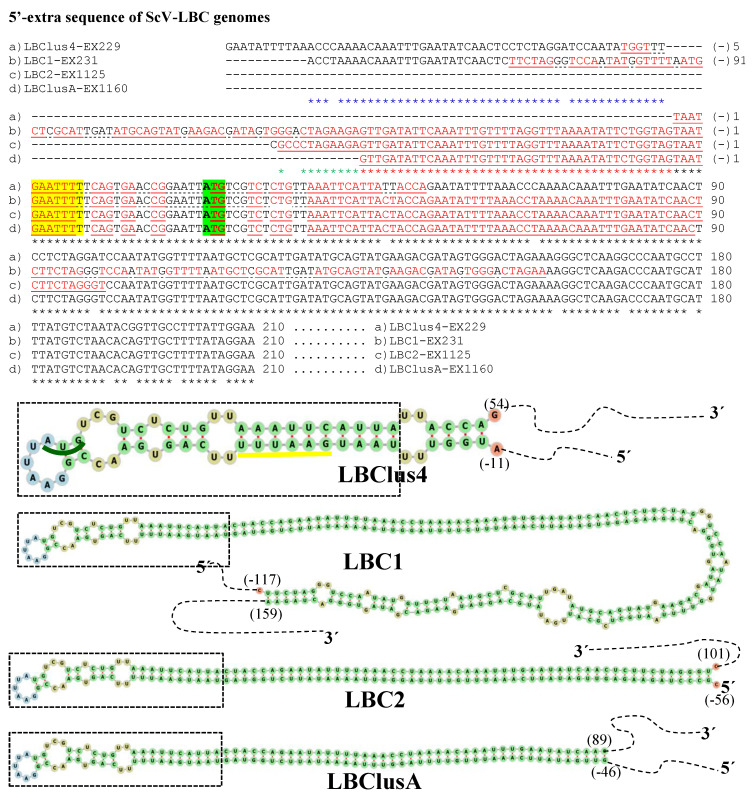
Nucleotide sequence (cDNA) alignment of 5′-extra sequences and proximal canonical sequences of ScV-LBC genomes from *S. cerevisiae*: (**a**) LBClus4-EX229, (**b**) LBC1-EX231, (**c**) LBC2-EX1125, and (**d**) LBClusA-EX1160. Black asterisks (*) indicate identical nucleotides in all genomes. Red asterisks (*) indicate identical nucleotides in (**b**) LBC1-EX231, (**c**) LBC2-EX1125, and (**d**) LBClusA-EX1160 genomes. Blue asterisks (*) indicate identical nucleotides in (**a**) LBClus4-EX229 and (**b**) LBC1-EX231 genomes. Green asterisks (*) indicate identical nucleotides in (**b**) LBC1-EX231 and (**c**) LBC2-EX1125 genomes. The 5′-GAATTTT of canonical sequences is yellow highlighted. The protein synthesis initiation codon of Gag-Pol is green shaded. Nucleotides of palindromic sequences are shown in red letters. Stem-loops are underlined. The unpaired nucleotides of each loop are dot underlined. The secondary RNA structures of possible 5′ stem-loops are shown at the bottom of the sequences.

**Figure 4:**
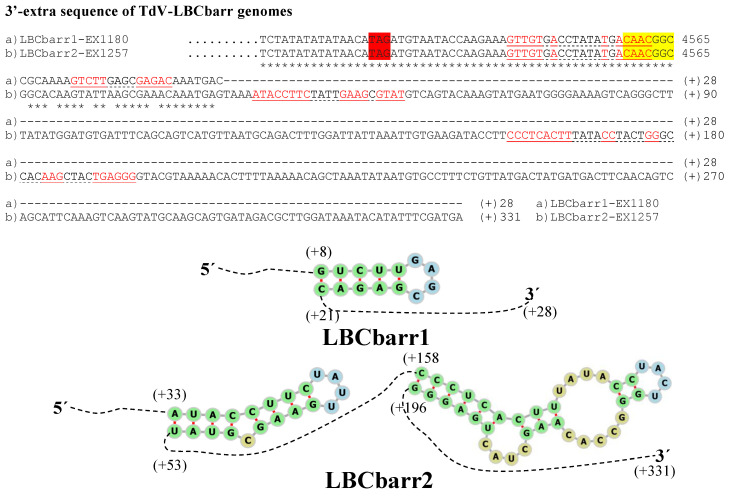
cDNA of 3′-extra sequences and proximal canonical sequences of TdV-LBCbarr1-EX1180 and TdV-LBCbarr2-EX1257 genomes. The CAACGGC-3′ ends of canonical sequences are highlighted. The protein synthesis stop codons of Gag-Pol are red-shaded. Nucleotides of palindromic sequences are shown in red. Stem-loops are underlined. The unpaired nucleotides of each loop are dot underlined. Asterisks (*) indicate identical nucleotides. The secondary RNA structures of possible 3′ stem-loops are shown at the bottom of the sequences.

**Figure 5:**
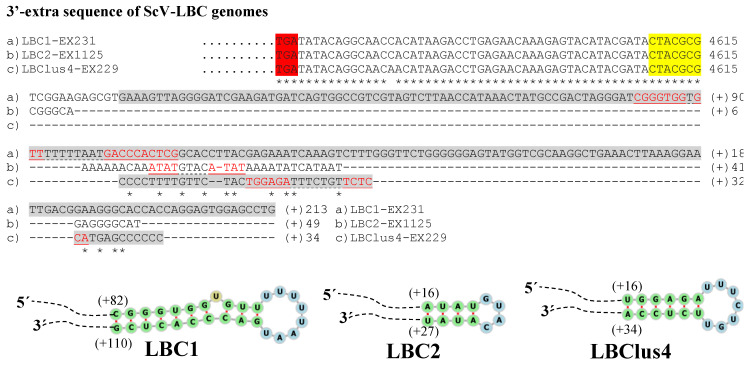
Nucleotide sequence (cDNA) alignment of 3′-extra sequences and proximal canonical sequences of ScV-LBC genomes: (**a**) LBC1-EX231, (**b**) LBC2-EX1125 and (**c**) LBClus4-EX229. Asterisks (*) indicate identical nucleotides. The CTACGCG-3′ ends of canonical sequences are yellow highlighted. The protein synthesis stop codons of Gag-Pol are red-shaded. The 3′-extra sequences identical to rRNA stretches (18S in LBC1-EX231, and 25S in LBClus4-EX229) are grey shaded. Nucleotides of palindromic sequences are shown in red. The stem-loops sequences are underlined. The unpaired nucleotides of each loop are dot underlined. The secondary RNA structure of possible 3′ stem-loops is shown at the bottom of the sequences.

The genome sequence obtained from the two Td-LBCbarr and the four Sc-LBC viruses was longer than the estimated canonical sequence (Table 1). Extra nucleotides were found beyond the 5′ and 3′ ends of the canonical genomes, in all cases except in the 3′-end of ScV-LBClusA. For sequence descriptions, nucleotides were numbered from the 5′GAATTT conserved motif in ScV-LBC viruses, which was considered as the 5′-end in the canonical genomes of *S. cerevisiae* LBC viruses [5,8,9]. The homologous motif 5′GAAATT was considered for TdV-LBCbarr genomes. The 5′-terminal G was considered as number 1. Additional nucleotides located upstream from the 5′GAATTT or 5′GAAATT motif were numbered with a negative symbol starting at (−)1 from the first nucleotide upstream from 5′G (Figure 2 and Figure 3). Similarly, additional nucleotides located downstream from the 3′-end of ScV-LBC (CTACGCG3′) [5] or TdV-LBCbarr1 (CCATAAGC3′) [9] genomes were numbered with a positive symbol starting at (+)1 from the first nucleotide located downstream from C3′ (Figure 4 and Figure 5).

No relevant identity was detected between the 5′- or 3′-extra sequences of V-LBC genomes from *T. delbrueckii* and *S. cerevisiae*. However, high local identity between the 5′- or 3′-extra sequences from Td-LBCbarr viruses, and between the 5′-extra sequences from Sc-LBC viruses was found (Figure 2, Figure 3 and Figure 4). No relevant identity between the 3′-extra sequences from Sc-LBC viruses was found (Figure 5).

A 173 nt region of TdV-LBCbarr1, most part in the 5′-extra sequence (170 nt) except 3 nt in the 5′-end [G(−)170–A3], was 100% identical to a homologous 173-nt stretch located near the 3′-end in the (−)RNA canonical sequence of the same genome [T31–C203 in the (+)RNA strand]. These stretches can form a stem-loop (ΔG = −1269 kJ/mol), with two contiguous loops, one of which contains as unpaired thee nucleotides of the 5′GAAATT conserved motif and the ATG start codon of the canonical sequence. A very similar stem-loop (ΔG = −1121 kJ/mol) was also found in TdV-LBCbarr2 (Table 1 and Figure 2). Moreover, a similar identity of 5′-extra sequence with part of the canonical sequence of (−)RNA strand, and possible stem-loop, was also found in the genomes of ScV-LBClus4 (ΔG = −86 kJ/mol) and ScV-LBClusA (ΔG = −327 kJ/mol). The 5′-extra sequence of ScV-LBC1 and ScV-LBC2 also showed identity with part of a canonical sequence, but in these cases, it was with the (+)RNA strand. Notwithstanding, a possible stem-loop was also found in these two genomes (ΔG = −604 kJ/mol and ΔG = −429 kJ/mol, respectively). Interestingly, all these four stem-loops also were very similar, containing four contiguous loops. The ATG start codon is also involved in one of these loops, as in TdV-LBCbarr genomes, but the 5′GAATTT conserved motif of ScV-LBC genomes is not (Table 1 and Figure 3). No probable kissing-loop interaction was found near the 5′-end of LBC genomes.

The 3′-extra sequences of TdV-LBCbarr1 and TdV-LBCbarr2 genomes also showed 100% identity to the (+)RNA strand (28 nt and 331 nt, respectively); and one (ΔG = −15 kJ/mol) and two (ΔG = −25 kJ/mol and ΔG = −39 kJ/mol) possible stem-loops were found, respectively (Table 1 and Figure 4). No identity to any known sequence was found for the 3′-extra sequence of ScV-LBC2 genome, but, again, a stem-loop can be formed (ΔG = −6 kJ/mol). The 3′-extra sequences of ScV-LBC1 and ScV-LBClus4 genomes showed 99% and 100% identity to part of the (+) strand 18S rRNA and (−) strand 18S rRNA (both of S. cerevisiae), respectively. Possible stem-loops were also found in these two cases, ΔG = −49 kJ/mol and ΔG = −30 kJ/mol, respectively (Table 1 and Figure 5). No intramolecular kissing-loop interaction was found near the 3′-end of LBC genomes.

## 4. Discussion

### 4.1. Canonical Nucleotide and Amino Acid Sequences of Td-LBC and Sc-LBC Viruses

Contrary to that found for comparison of most L viruses, nucleotide sequence identity was lower than amino acid sequence identity when comparing TdV-LBC and ScV-LBC. This is mainly because TdV-LBCbarr Gag-Pol contains two stretches that share very low sequence identity with the homologous region of ScV-LBC: LIS I and LIS II. It has been described in various viral genomes that frameshifting due to nucleotide insertions or deletions (indels), which may cause the premature termination of protein synthesis, can be restored to produce functional proteins by a secondary indel near the primary indel site. This phenomenon is known as “compensatory frameshift” [32], and may explain the low amino acid identity in LIS I and II. These LIS regions could be the result of successive indel followed by compensatory frameshift events. This way, changes in amino acid sequence would be more relevant than changes in nucleotide sequence. As consequence, these indels will decrease Gag-Pol identity while somehow maintaining the nucleotide sequence identity between the RNA regions that belong to the LIS of these viruses. This would somehow allow LBC viruses to maintain specific functions of the (+) RNA while changing specific functions of the variable stretches of Gag-Pol; such as gaining the ability to bind newly emerged versions of M (+) RNA for packaging into the TdV-LBCbarr2 virion, or losing the ability to bind M (+) RNA as may have occurred in ScV-LBC. This will always require restoring the correct translation frame of the RdRp domain by “compensatory frameshift”. However, eventually, the correct translation frame may also be temporarily restored by the involvement of the second putative slippery site “GGGGAG”, which is followed by a putative in-frame translation re-initiation codon and is located upstream from Pol domains in all LBC genomes. This strategy would temporarily ensure the availability of active RNA polymerase with the correct amino acid sequence of the RdRp domain. Meanwhile, a subsequent indel may occur to get the definitive “compensatory frameshift”. This strategy may not be required to temporarily compensate indels in LIS II, since there are no Pol essential domains downstream from this stretch. Alternatively, the presence of a second putative in-frame translation re-initiation codon downstream from the Gag stop codon raises the possibility that some amount of free (unattached to Gag protein) RdRp could be synthesized as a functional enzyme.

Something similar may occur for the 42-aa variable region found in the Gag-Pol encoded by LA viruses. When comparing only this region of TdV-LAbarr1 and ScV-LA1-original, the amino acid identity (20%) was much lower than nucleotide identity (44%) [14]; which is similar to that found between TdV-LBCbarr LIS and the homologous stretch of ScV-LBC. Although it has been suggested that this 42-aa variable region is indeed separating the two domains of LA Gag-Pol, and does not interact tightly with other amino acids of Gag and Pol domains [15], it could be involved in a more relevant function than previously thought. Thus, since this region contains many hydrophobic amino acids, it could be involved in shaping the Gag-Pol domain responsible for the specific recognition of the (+)RNA to be packaged in the virion. Contrastingly, the second putative slippery site, which could allow temporary restoration of the correct translation frame, has not been found in the LA virus. Notwithstanding, a second putative in-frame translation re-initiation codon located downstream from the Gag stop codon was also found in all LA genomes; which also raises the possibility that some amount of free RdRp could be synthesized [14].

Some stretches of TdV-LBCbarr genomes showed relevant identity with the mitochondrial chromosome of *C. tenuimanus*, and some genomic sequences of *P. xenopodis*, *H. sapiens*, *V. vinifera*, *Marinilactibacillus* sp., *S. fibuligera*, and *S. scrofa*. All these nucleotide stretches coincide with rather conserved amino acid sequences of Gag-Pol (Figure 1). These findings suggest the transfer of xenolog RNA stretches from different organisms to the LBC virus genome, maybe by recombination with the viral mRNAs, as previously suggested [17]. This process could have occurred during the phylogenetic appearance of L viruses. A different evolution of *S. cerevisiae* L viruses with respect to *T. delbrueckii* may explain why the relevant identity of the xenolog sequences with the *S. cerevisiae* genome was not detected. Only a similar stretch was found in an Sc-LBC virus (ScV-LBC2-EX1125) that showed relevant identity with a 5′-extra sequence found in ScV-M1-EX231 [4]. This result suggests that V-L and V-M RNA could recombine if they coincide in the same yeast strain, as previously suggested [4]. However, the coincidence of ScV-LBC2 and ScV-M1 in the same yeast strain has not yet been found. Despite this, ScV-LBC2 and ScV-LBC1 share 96% identity [9]; which suggests that either of these two helper viruses could maintain ScV-M1 or ScV-M2 in a K1 or in a K2 killer yeast, respectively.

### 4.2. Features Found in the 5′- and 3′-Extra Sequences from LBC Genomes

The additional sequences that we have found in V-LBC genomes may be only a part of the completed extra sequences of each virus. It cannot rule out that the shorter extra sequences might just reflect some lack of robustness during the contig assembling of some samples. We do not know whether these extra sequences are present in the dsRNA located inside of the virion or they are only a part of a viral RNA intermediary. Similar results were found for yeast V-M and V-LA genomes. However, identity between extra and canonical sequences of the same genome was only found in V-LBC as well as in LA viruses, but not in M viruses [4,14]. The matching base pairs of these sequence stretches to allow the formation of double-strand stem-loops at the ssRNA ends of these viruses, which may protect viral ssRNA from single-strand exonucleases. These stem-loops may also provide a free 3′-end that could be used as a primer by RNA-dependent RNA polymerases for dsRNA synthesis or for mRNA transcription. As previously suggested [14], these stem-loops could even have more than one function. In addition, intramolecular interaction between extra sequences and proximal canonical sequences may also play a yet unknown role in the biology of these L viruses [33]. Although LBC 5′ stem-loops resemble that previously found in TdV-LAbarr1, no kissing-loop interaction involving the 5′-end of LBC genomes was detected, as it was previously found in LAbarr1 and ScV-LA1 genomes. Therefore, unlike previously proposed for LA viruses [14], we cannot suggest a possible role of kissing-loop interactions in favoring RNA polymerase access to the (−)RNA strand for mRNA transcription in LBC viruses.

Ribosomal RNA stretches were detected in the 3′-extra sequences of ScV-LBC1 and ScV-LBClus4, but not in TdV-LBCbarr extra sequences. Similarly, the 3′-extra sequences of several M viruses from *S, cerevisiae*, and *T. delbrueckii* also contain rRNA sequences [4], as well as the 5′- and 3′-extra sequences of several ScV-LA genomes [14]. Strikingly, all these sequences so far found in L and M viruses belong to different regions of the same rRNA or to different types of rRNA. Accordingly, these rRNA stretches do not share a relevant identity. It has been suggested that ScV-M RNA could bind to other RNAs from the host or from other viruses [4], similarly to that suggested for poliovirus RNA [34] and plant viruses [35]. It is thus possible that M and L viruses integrate into cellular RNA as rRNA, as retroviruses and retrotransposons do in chromosomal DNA. This strategy may protect these viruses from disappearance if some copies of their genome remain attached to a more persistent RNA. It has even been suggested that yeast viruses could recombine with rRNA and form a ribonucleoprotein resembling the yeast ribosome. The formation of these ribosome-like complexes may ensure that the yeast virus remains in the cell [14]. This could be similar to the endogenization of some ant RNA virus genomes involving nuclear chromosomes [36], but using a different strategy that involves rRNA in yeasts. Untangling the roles of the rRNA sequences located in 5′ and 3′ extra sequences of V-L and V-M genomes could reveal complex biological functions. Indeed, some rRNA-containing mRNA sequences have been described in mammalian cells. Some of these rRNA sequences appear to function as cis-regulatory elements involved in translation efficiency, while other sequences seem to be involved in some neurodegenerative diseases [37,38,39].

The finding of identical sequence stretches in the 5′-extra sequences of all LBC genomes, the 3′-extra sequences of LBCbarr genomes, and the 5′-extra sequences of some ScV-LA genomes [14], indicates that these extra sequences could have a common origin. As these extra sequences often show relevant local identity with some stretches of the canonical sequences, it has been suggested that they may originate from an imprecise molecular mechanism involved in viral replication [14], such as cap-snatching [40].

## 5. Conclusions

The two LIS found in TdV-LBCbarr Gag-Pol may have been originated by successive indels that allow virus speciation while maintaining the fundamental functions of (+) RNA, and Gag and Pol domains. The existence of a second in-frame translation re-initiation codon, preceded by a possible slippery site, may facilitate a required “compensatory frameshift”. This strategy would allow LBC viruses to change their ability to bind newly arisen versions of M (+)RNA for packaging and replication. The transfer of xenolog RNA sequence stretches from different organisms to the canonical sequence of V-L genomes could be at the inception of these viruses. The extra sequences located at both sides of V-LBC canonical genomes may form RNA secondary structures involved in avoiding ssRNA degradation and facilitating dsRNA synthesis, or in a still unknown biological function related to virus replication. The finding of rRNA stretches in the 3′-extra sequences of ScV-LBC genomes may be a consequence of recombination of virus RNA with yeast rRNA. This could form a kind of ribonucleoprotein that somehow resembles the yeast ribosome and ensure the permanence of these viruses in the yeast cell.

## Figures and Tables

**Table 1:** Yeast strains and characteristics of the V-LBC genomes sequenced by HTS.

Yeast Strain	Killer Phenotype/LBC dsRNA Type	Sequenced Length (bp) (Canonical)	5′-Extra Sequence (bp)/% Identity to, Size [Position]	3′-Extra Sequence (bp)/% Identity to, Size [Position]	Stem-Loop Involving 5′-Extra Sequence [Position] (ΔG)	Stem-Loop Involving 3′-Extra Sequence [Position] (ΔG)
Sc EX231	K1/LBCM1-1	4971 (4615)	143/100% identity to (+) strand, 95 nt [A(−)143–A(−)49] to 95 nt [A65–A159]	213/99% to (+) strand Sc 18S rRNA, 201 nt [G(+)13–G(+)213]	[T(−)116–A159] (−604)	[C(+)82–G(+)110] (−49)
Sc EX1125	K2/LBCM2-4	4722 (4615)	58/100% identity to (−) strand, 62 nt [C(−)56–T6] to 62 nt [A38–G99]	49/no identity found	[G(−)55–T100] (−429)	[A(+)16–T(+)27] (−6)
Sc EX229	Klus/LBCMlus4	4839 (4615)	63/100% identity to (+) strand, 64 nt [G(−)63–G1] to 64 nt [G54–G116]	43/100% to (−) strand Sc 25S rRNA, 43 nt [C(+)2–C(+)44]	[T(−)10–A53] (−86)	[T(+)15–A(+)34] (−30)
Sc EX1160	Klus/LBCMlusA	4661 (4615)	46/100% identity to (−) strand, 52 nt [G(−)46–T5] to 52 nt [A38–C89]	None	[G(−)46–C89] (−327)	None
Td EX1180	Kbarr1/LBCbarr1	4763(4565)	170/100% identity to (−) strand, 173 nt [G(−)170–A3] to 173 nt [T31–C203]	28/100% identity to (+) strand, 28 nt [C(+)1–C(+)28] to 28 nt [C4466–C4493]	[G(−)170–C203] (−1269)	[G(+)8–C(+)21] (−15)
Td EX1257	Kbarr2/LBCbarr2	5115 (4565)	219/100% identity to (−) strand, 151 nt [C(−)148–A3] to 151 nt [T31–G181]	331/100% identity to (+) strand, 22 nt [A(+)7–A(+)28] to 22 nt [A4471–A4492]; and 307 nt [T(+)25–A(+)331] to 307 nt [T3175–A3481]	[C(−)148–G181] (−1121)	[A(+)33–T(+)53] (−25)[C(+)158–G(+)196] (−39)

ΔG for the (+)ssRNA is in kJ/mol, and it was obtained with the program MFOLD. Nucleotide numbers refer to the RNA (+) strand of the viral genome. *Sc*, *Saccharomyces cerevisiae*. *Td*, *Torulaspora delbrueckii*.

**Table 2:** Nucleotide sequence of yeast LBC and LA virus genomes used in this study.

Virus-Yeast Strain	Accession Number	Reference/Comment
TdV-LBCbarr1-EX1180	OL469171	[9]
TdV-LBCbarr2-EX1257	OL469172	[9]
ScV-LBC1-original	U01060.1	[8]. Previously known as ScV-La or L-B-C. Here renamed to distinguish it from other LBC viruses from different K1 killer strains
ScV-LBClus4-EX229	KT784813.1	[5]
ScV-LBC2-S3920	KX906605.1	[5]
ScV-LBC1-EX231	OL469175	[9]
ScV-LBC2-EX1125	OL469176	[9]
ScV-LBClusA-EX1160	OL469174	[9]
ScV-LBClus4-EX229	OL469173	[9]
TdV-LAbarr1-EX1180	MW174763	[14]
ScV-LA1-original	J04692.1	[13]. Previously known as ScV-LA. Here renamed to distinguish it from other LA viruses from different K1 killer strains
ScV-LA1-EX231	MW174760	[14]
ScV-LAlus4-EX229	MW174758	[14]
ScV-LAlus1-EX436	MW174761	[14]
ScV-LAlusA-EX1160	MW174762	[14]
ScV-LA2-S3920	KC677754.1	[5]
ScV-LA2-EX1125	MW174759	[14]
SpV-LA28	KU845301.2	[30]. Previously assigned to *S. cerevisiae* but thereafter re-assigned to *S. paradoxus* [7]

Sc, *Saccharomyces cerevisiae*. Td, *Torulaspora delbrueckii*. Sp, *Saccharomyces paradoxus*.

## Data Availability

Not applicable.

## Supplementary Materials

The following supporting information can be downloaded at: https://www.mdpi.com/article/10.3390/microorganisms10010173/s1, Figure S1: Multiple sequence alignment between ScV-LBC1-original, ScV-LBClus4, and TdV-LBCbarr2 (+) strand nucleotide sequences (cDNA). 5′GAA(A/T)TT conserved motif (5′ conserved), translation initiation (start of Gag and Gag-Pol, or internal possible start ATG in Pol ORF of LBC1-original, LBClus4 and LBCbarr2), termination codons (stop of Gag and stop of Gag-Pol), ribosome frameshifting site (−1 frameshift site), frameshifting associated sequence (stem loop for frameshift), packaging signal (stem loop for packaging), and replication signal (stem loop for replication) are indicated, shaded and/or underlined in the nucleotide sequence. The highly conserved RdRp domain located in the central third of Pol is also underlined. , ribosomal frameshift. Asterisks (*) indicates identical nucleotide positions. Sequence stretches of V-LBC genomes sharing high local identity with some mitochondrial or genomic sequences of several organisms are brown shaded, the sequences of other organisms are shown above each homologous stretch of LBC genome, and each percentage of identity is in parenthesis.
