# Supplementary material for: New Insights into the Genome Organization of Yeast Double-Stranded RNA LBC Viruses
Source: Microorganisms. 2022 Jan 14;10(1):173. doi: 10.3390/microorganisms10010173 (PMC8780742; doi:10.3390/microorganisms10010173)
Supplement: Supplementary file 1 [file microorganisms-10-00173-s001.zip › microorganisms-1546329-supplementary.pdf]

# *Supplementary Materials*

## 1.1 Supplementary Figures

```
LBCbarr2 GAAATTAGCTGTAGCCAATCGATTGCCATGTTCTCAACTCTTAACAATTTACTACCCGAT 60
LBC-ori  GAATTTTTC----GGTGAACCGGAATTATGTCGTCTCTGTTAAATTCATTACTACCAGAA 56
LBClus4  GAATTTTTC----AGTGAACCGGAATTATGTCGTCTCTGTTAAATTCATTATTACCAGAA 56
         *** **  *        * *       ****  **     * **    *** **** **
        5' conserved            Start of Gag and Gag-Pol
LBCbarr2 ACTTTAAACCCTCGTAAGAATTTCAAACTTACTCACCATAGAATTAAATATGGTATGAAT 120
LBC-ori  TATTTTAAACCTAAAACTAATTTGAATATCAACTCTTCTAGGGTCCAATATGGCTTTAAT 116
LBClus4  TATTTTAAACCCAAAACAAATTTGAATATCAACTCCTCTAGGATCCAATATGGTTTTAAT 116
           *** ** **    *  ***** **  * *       ***  *  *******  * ***

LBCbarr2 GCCAACATCAATTTCGATGTTTCTGATGACTTTGGGAAATATGAAGGTAATAGACCGGGT 180
LBC-ori  GCTCGCATTGATATGCAGTATGAAGACGATAGTGGGACTAGAAAAGGCTCAAGACCCAAT 176
LBClus4  GCTCGCATTGATATGCAGTATGAAGACGATAGTGGGACTAGAAAGGGCTCAAGGCCCAAT 176
         **   ***  ** *  *   *   ** **   *****      * **    ** **   *

LBCbarr2 GCTTTTATTGGAAATGAAGTATCTCTTTATGGTAATTACAATTCTTATATCACGGATGAC 240
LBC-ori  GCATTTATGTCTAACACAGTTGCTTTTATAGGAAACTATGAAGGTATTATTGTTGATGAC 236
LBClus4  GCCTTTATGTCTAATACGGTTGCCTTTATTGGAAACTATGAAGGTGTCATCGTTGATGAC 236
         ** *****    **    **  *  **   ** ** **  *   *   **    ******

LBCbarr2 GTGCCAATCTATGATGGTATGCGGTCTGATGTGATCACTCCTGAAGGAGAGTTCAGCCAT 300
LBC-ori  ATTCCGATATTGGATGGTCTTAGGGCCGACATTTTTGATACTCATGGTGACTTAGACATG 296
LBClus4  ATCCCGATATTGGATGGCCTTAGAGCTGACATTTTTGATACTCATGGTGATTTAGACATG 296
          * ** ** *  *****  *  *  * **  *  *   * ** * ** ** **   *

LBCbarr2 GAATTAGCTGAGCAAGTGTTAAGTAAAGCGACATCGGACCGAGCATCCGCTCAGAAGATC 360
LBC-ori  GGCCTCGTTGAGGATGCATTGTCTAAGAGTACCATGATTAGAAGGAATGTACCAACTTAT 356
LBClus4  GGCCTCGTAGAGGATGCATTGTCCAAAAGTACTATGATTAGAAGGAATGTACCAACTTAT 356
         *   * *  *** * *  **    **    **   *    **      *  *  *

LBCbarr2 ACGCAATACTTGGCATACAATGGAGGTATGCGCAATTGTGTATCTATATTGTATAACTTA 420
LBC-ori  ACTGCTTACGCGAGTGAATTACTGTATAAGAGAAACCTTACATCTCTATTTTACAATATG 416
LBClus4  ACTGCTTACGCGAGTGAATTATTGTATAAGAGAAACTTAACATCTCTGTTTTACAATATG 416
         **    ***  *    *         ** * * **      **** * ** ** **  *

LBCbarr2 CTGCGTATGTATGTCATGAATGAGTTTGGCGAGGTGGTCTATGACCCAAACGAGATATTC 480
LBC-ori  CTCCGTTTATACTACATTAAAAAATGGGGCAGTATTAAGTATGAAAAAGATGCCATCTTT 476
LBClus4  CTCCGTTTATATTACATTAAAAAATGGGGTAGTATTAAGTATGAAAAAGATGCCATTTTT 476
         ** *** * **   *** **  * *  **     *    *****   * * *  ** **

LBCbarr2 TACGACAATGGACATGTGTGTATTAAAAATCATCAGATGTTTCCAAAACATCTAGAGAAG 540
LBC-ori  TATGATAATGGCCACGCCTGTCTTTTAAACAGGCAATTGTTTCCAAAGTCTCGTGATGCT 536
LBClus4  TATGATAATGGCCACGCCTGTCTTTTAAACAGGCAATTGTTTCCGAAGTCTCGTGATGCT 536
         ** ** ***** ** *  *** **  ***    **  ******* **   **  **

LBCbarr2 AAGTCATTAGCTAAGGCTGTT---GAGTACACTCCTGCTATGATCGAAGATGCTAGCACC 597
LBC-ori  TCTTTGGAATCAAGCCTCTCTTTGCCTGAGGCTGAAATTGCAATGCTTGATCCTGGCCTG 596
LBClus4  TCTTTGGAATCAAGCCTCTCTTTACCTGAGGCAGAAATTGCAATGCTTGATCCTGGATTG 596
            *    * * *       *       *  *      *   **    *** ** *

LBCbarr2 TTATGGCGCGAAGGTGACATACCTGAATGTCCTTCATTGATTTGGACAGGATTTATGCGT 657
LBC-ori  GAATTTCCAGAAGAGGATGTGCCTGC---------AATTTTATGGCACGGCAGAGTGTCA 647
LBClus4  GAATTCCCAGAAGAGGATGTGCCTGC---------AATTTTATGGCATGGCAGAGTGTCA 647
           **  *  ****  **  * ****          * *  * ***   **      **

LBCbarr2 GATAAGCAAGCTACTATCGTTTCACGGGCAGCAGGTGGATTCATTAACAGAGCACCTTTC 717
LBC-ori  TCCAGAGCAACGTGTATCTTAGGGCAAGCTTGCTCAGAGTTCGCGCCTCTGGCCCCCTTT 707
LBClus4  TCCAGAGCTACGTGTATCTTAGGGCAAGCCTGCTCTGAATTTGCGCCTCTGGCCCCCTTT 707
            *      *   **** *    *  **       *  **          ** ** **

LBCbarr2 GGAGTAGCTCATGAGTCAAAAAATCTTACTGAAAGATTAGGTATCATGGCAACTGGAGGA 777
LBC-ori  TCGATTGCGCATTATTCACCACAATTGACGAGAAAACTATTTGTCAATGCGCCCGCTGGG 767
LBClus4  TCAATTGCGCATTATTCACCACAATTGACAAGAAAACTATACGTCAATGCGCCCGCTGGG 767
             * ** *** * ***  * *  * **   ** * **    ***  **  * *  **
```

```
LBCbarr2 CTCTCTAATGTAGAAGATTATAACTATGGTCCTTCTGATATATTGTTCGTCCTTAACAAG 837
LBC-ori  ATTGAGCCTA---GCTCCGGGCGGTATACTCACGAGGATGTAAAAGATGCGATTACGATC 824
LBClus4  ATTGAGCCTA---GCTCCGGGCGGTATACTCACGAGGATGTAAAAAATGCGATTACGATC 824
          *      *               ***  **     *** **      *   ***  *

LBCbarr2 CTGGTGACAGATAACGGGCTTTACACTGACTTCACTTACGCTTATCTAATGTTGTTACAA 897
LBC-ori  CTTGTGTCTGCAAACCAGGCTTATACTGACTTTGAAGCAGCATACTTGATGCTTGCTCAA 884
LBClus4  CTTGTGTCTGCTAACCAGGCTTATACTGACTTTGAGGCAGCATACTTGATGCTTGCTCAA 884
         ** *** * *  ***  *  *** ********       ** **  * *** *    ***
                        ATACCAAGGTCTACTGAAGCTCCAGCATGG
LBCbarr2 GTATTAGTGTCTCCTATACCAAGGTCTGCTGAAGCTGCAGCATGGTTCGTCGGTGTCCAG 957
LBC-ori  ACGTTGGTCTCACCTGTACCACGCACTGCCGAAGCAAGTGCATGGTTCATCAATGCTGGC 944
LBClus4  ACATTGGTCTCACCTGTACCACGCACAGCCGAAGCTAGTGCATGGTTCATTAATGCTGGT 944
            ** ** ** *** ***** *  * ** *****    ********* *   **
                Mitochondrial cytochrome oxidase I of Cherax tenuimanus (93%)
LBCbarr2 ACAGTGTCTTTACCTAGGTTGGTGACTGACACAGGAAACATACCACAGTTATATCAGGGG 1017
LBC-ori  ATGGTCAATATGCCAACTTTGTCATGTGCAAATGGTTATTATCCAGCACTGACCAATGTC 1004
LBClus4  ATTGTTAACATGCCTATCTTGTCATGTGCTAATGGCTACTACCCAGCCTTAACAAATGTT 1004
         *  **     * ** *  ***     **  *  **  *    ***    *     * *

LBCbarr2 AAAGTCTATGCTAGGTTTAATGACTGGAAAGAGACTATGGCTAACTTCAGAGCATATCCT 1077
LBC-ori  AATCCTTACCACCGGCTAGACACATGGAAAGATACGTTAAATCATTGGGTGGCTTATCCC 1064
LBClus4  AATCCTTACCACCGGTTAGACACATGGAAAGACACTTTAAATCATTGGGTGTCTTACCCC 1064
         **    **     ** *  *    ******** **  *   * * *      * ** **

LBCbarr2 AAGTCATGTTTATTGCATAGTATTGTAGTGAATGAAGCTGTTTACGTTGAATTGAACAAT 1137
LBC-ori  GACATGCTGTTTTACCATTCAGTGGCAATGATTGAGAGCTGCTATGTTGAACTCGGGAAT 1124
LBClus4  GACATGTTATTTTACCATTCGGTGGCAATGGTTGAGAGCTGTTATGTTGAACTGGGGAAT 1124
          *       ** *  ***    * * * **  ***       ** ****** *    ***

LBCbarr2 CTTAACAGAGTGAGTCCTTTTGATGCAATTAATCGAGAAAATTTTGTTGAGGTAGCTGTA 1197
LBC-ori  GTGGCTCGTGTGTCAGACAGTGATGCAATAAACAAATACACTTTCACTGAGCTATCAGTG 1184
LBClus4  GTCGCACGTGTGTCAGACAGTGATGTAATTAACAAGTATACCTTCACTGAGCTATCTGTG 1184
          *     * ***        ***** *** **     * *  **   **** ** * **

LBCbarr2 CAAGGGCAGCCTGTCACAATGGGCGGGATATTGAATGATCTGACTTTAGTGTCGATGCGG 1257
LBC-ori  CAAGGACGGCCTGTTATGAATCGAGGAATTATTGTAGATCTGACACTTGTGGCAATGCGT 1244
LBClus4  CAAGGCCGGCCTGTTATGAACCGAGGGATTATTGTTGACTTGACACTTGTAGCAATGCGT 1244
         ***** * ****** *  *   * ** **  *    **  ****  * **  * *****

LBCbarr2 TATTCTAAGGAATTTGCACTGGCATATCCTGTGTCCACAGGACTTACAAGGACTAACATT 1317
LBC-ori  ACTGGTAGGGAGATCTCACTACCTTACCCGGTCAGCTGTGGCCTGACCCGTACAGACGCG 1304
LBClus4  ACTGGTAGAGAGATCTCACTACCTTACCCAGTTAGTTGTGGTTTGACCCGTACAGACGCA 1304
           *  **  **  *  ****  * ** ** **       **  * **  * **  **

LBCbarr2 ATGGCACAAGAAATAGCTCTTGAGGTACCAATAACAGTAAAAGACCTAGATGCACCTAAA 1377
LBC-ori  TTATTGCAAGGTACTGAGATTCACGTTCCAGTTGTTGTCAAAGATATCGACATGCCCCAG 1364
LBClus4  TTATTGCAAGGTACGGAGATTCATGTTCCAGTTGTCGTCAAAGACATGGACATGCCTCAG 1364
          *    ****  *  *   ** * ** *** *    ** *****  * **    **  *

LBCbarr2 TATTACAACATTGCAGAACCTATTATAACCGCAAATCAACATGCAACACTCATTGTTACT 1437
LBC-ori  TATTACAACGCGATTGATAAGGATGTTATTGAGGGGCAGGAAACTGTGATTAAAGTGAAA 1424
LBClus4  TATTATAATGCAATCGATAAGGATGTTATTGAAGGGCAAGAAACTGTAATAAAGGTGAAG 1424
         ***** **       **      * * *  *     **  *  *     * *  ** *

LBCbarr2 AATATGACGGCACCTTTGTATCCAGTAATGTCTTACGGGATTAATACTGATGACTATTAT 1497
LBC-ori  CAGCTGCCACCAGCTATGTATCCAATTTATACTTACGGGATCAACACTACTGAATTCTAT 1484
LBClus4  CAACTGCCACCAGCAATGTATCCGATTTATACTTATGGTATCAACACTACAGAGTTTTAC 1484
          *  ** *  ** *  *******  *     **** ** ** ** ***   ** *  **

LBCbarr2 TGTAACGATACGAACATCAGTATCAAATTTA---AGGCACCTGTGACTGCAAGTGGGATA 1554
LBC-ori  TCTGACCATTTTGAAGACCAGGTACAAGTTGAAATGGCACCAATCGATAATGGAAAAGCA 1544
LBClus4  TCCGACCATTTTGAGGACCAGGTACAAGTAGAAATGGCTCCTATCGATAATGGGAAGGCA 1544
         *   ** **    *   *    *  ** *      *** **  *   *    *      *
                                    AAGTTTATGAATGT^GTATGCGTATGTGTGGGCAC
LBCbarr2 TCTTTTACAGAAGCAGAAAATTTCTCAAAGTTTATGAACGTTATGCGTATGTGTGGGTAT 1614
LBC-ori  GTTTTTAACGATGCAAGAAAGTTTTCGAAATTTATGTCCATAATGCGCATGATGGGGAAT 1604
LBClus4  GTCTTTAATGATGCTCGAAAATTCTCTAAATTCATGTCTATAATGCGCATGATGGGGAAT 1604
            ****  ** **   *** ** ** ** ** ***    * ***** ***   *** **
         AAT           Protopolystoma xenopodis, Xenopus parasitic worm (95%)
LBCbarr2 AACGTTGAGGCCAGAGAGATGTTTTCAGGCAAGACTGTCATTAACTGGGCAGATAATGCT 1674
LBC-ori  GATGTTACTGCTACTGATTTAGTTACAGGTAGAAAAGTGTCGAATTGGGCCGACAACTCA 1664
LBClus4  GATGTTACGGCAACTGATTTAGTAACAGGTAGAAAGGTGTCGAATTGGGCCGACAACTCA 1664
```

```
          * ***    ** *   **   *   *   **** *   *   **     ** ***** ** **   *

LBCbarr2 AGTGGAAGATTTTTATATACAGATGCTCAAGTTGGTGAGGATATACATTATCATGTACCT 1734
LBC-ori  TCAGGGCGTTTCTTGTACACGGATGTGAAGTATGAAGGACAAACTGCCTTTTTGGTTGAT 1724
LBClus4  TCAGGGCGTTTCTTATACTCAGATGTCAAGTATGAAGGCCAAACTGCTTTTTTGGTTGAT 1724
            **  * ** ** **  * ****    *    **   *    * *      * *    **    *

LBCbarr2 TACGCCAACATTCATAGGCGGAGTAATTCATGGTTAAACGTGATAACCTTTAGAGGTGAA 1794
LBC-ori  ATGGATACTGTCAAGGCGAGAGACCACTGTTGGGTGTCAATTGTTGATCCTAATGGTACA 1784
LBClus4  ATGGACACTATTAAGGCTAGAGACCATTGCTGGGTGTCAATTGTGGATCCTAATGGCACG 1784
               *   *    *  *      *      * *  *** *      *   *       **  **

LBCbarr2 ACAGCCTTCAATGCGAACATCACAGCTGTGAATTTTAAAATGTTCAACGGCCCATCTGAA 1854
LBC-ori  ATGAACTTGTCATATAAGATGACCAATTTTAGAGCAGCAATGTTTTCTAGAAACAAGCCC 1844
LBClus4  ATGAGCTTGTCATATAAGATGACAAATTTCAGGGCAGCAATGTTTTCAAGAAACAAACCT 1844
         *     ***       ** ** **    * * *        ******      *

LBCbarr2 GTACTAATGACAGGGCCTCGAGTTATGCCAGTACAGATGAATGACTTCGTAGACAAGGCA 1914
LBC-ori  TTGTATATGACAGGGGGGTCAGTCAGGACCATAGCTACTGGCAATTATCGAGATGCTGCT 1904
LBClus4  CTATACATGACAGGTGGATCAGTCAGGACTATAGCTACTGGTAACTACCGTGATGCCGCT 1904
          *     ********       *** * * *  **    *       * *      **     **

LBCbarr2 ATTGAAATTGCTTCTCGTACTGACACGATTAAAATCTTACCTCTACGGAAAACTATGCTG 1974
LBC-ori  GAAAGATTACGTGCAATGGATGAAACGCTCAGATTAAAACCTTTTAAGATTACTGAGAAG 1964
LBClus4  GAGAGGTTGCGTGCAATGGACGAAACGTTGAGGTTAAAGCCTTTTAAGATTACTGAGAAG 1964
                *   * *        ** *** * *    *     *** *    **   ***  *  *
            ∩
LBCbarr2 CCGGATTTTCGATTCCTCAGGTATCAGTTCCCGGGTGTGTCGGGGATGGGGAATACTGTC 2034
LBC-ori  TTGGATTTTCGTGTAGCAGCTTACGCGATACCAAGTTTGTCGGGCAGCAATATGCCATCC 2024
LBClus4  TTGGATTTTCGTGTAGCAGCTTATGCGATACCAAGTCTGTCGGGCAGCAATATGCCATCC 2024
         *********  *       **    * * **   ** ******* *      *   *    *
          -1 Frame-shift site   Stem-loop for frame-shift (ΔG=-21.9 kJ/mol in LBCbarr2, ΔG=-35.1 kJ/mol in LBC-ori)
LBCbarr2 TTGCCACGGGAGAGCACACAACAGTTTGGCCAGGAAGAACAAGAGCTAGTGAATCCAGAG 2094
LBC-ori  TTACACCATCAGGAACAACTACAGATATCAGAAGTGGACGCGGAACCAATCAATCCTATA 2084
LBClus4  TCACACCATCAGGAACAACTACAGATATCAGAAGCGGACGCGGAGCCGATCAATCCTGTG 2084
         *  *  *   **     ** **** *     * *  **     ** *   * *****

LBCbarr2 GCGAGAGATATGTCAATCCAGGATGTAGAGTAG-------------TACCGACATATCTA 2141
LBC-ori  GGAGAGGACGAACTTCCACCGGATATAGAATAGGTGTCGAAGACGATGAGGACTTAGATA 2144
LBClus4  GGAGAGGACGAACTTCCACCGGATGTAGAGTAGGTGTTGAAGATGATGAAGACTTAGATA 2144
         *     **          * **** **** ***             *   *** **   **
                                Stop of Gap LBC and LBCbarr
LBCbarr2 A----------CAATAGAC-------------------GGG---GAGATGACGACAACGC 2169
LBC-ori  TTGGTACGGTCAAATACATTGTGCCATTGTATTTGAACGGTGATAATGTGGCACAAAATT 2204
LBClus4  TTGGTACGGTCAAATACATTGTGCCATTGTATTTGAACGGTGATAATGTGGCACAAAACT 2204
                     **** *                   **     *  ** *    **
                                            Possible frame-shift site  Possible start of free LBCbarr2 RdRp
LBCbarr2 CAGAGGAAGCTGACAAAGTGCT---TCTGGCCATGTATTATTCAAACCAGAAAGTGATTA 2226
LBC-ori  GTTTAGAAGCAACACACGTGCTTATCAAAGCTTGTAGTATTGCGAACCGGATTGTAGATG 2264
LBClus4  GTTTAGAAGCAACACATGTACTTATTAAAGCTTGTAGTGTTGCAAACCGGATTGTAGATG 2264
              *****     * ** **        **       *   * * **** **   **    *
        Stem-loop (ΔG=-26.1 kJ/mol)
LBCbarr2 AGACTAAGGCAGGATCTTTCACTCAGACTGGATTATCTAAGCAGATGATAGTTCATAATG 2286
LBC-ori  ACGGAGAGGGTCACTGTTTCACACAGCAAGGGCTGGCGCAGCAGTGGATCTTCCATAGGG 2324
LBClus4  ATGGAGAGGGTCACTGCTTTACACAGCAAGGGCTAGCACAGCAGTGGATCTTCCATAGGG 2324
         *     ***     *  ** ** ***    **  *   *   *****  ***   * ****   *

LBCbarr2 GGATGACAGTTAATACCGACGTCATCAGAATTGGGGATGTCAATTGTTATTATGTACGGT 2346
LBC-ori  GGGAGATGATATTTGTGAAGGCGGTACGCATTGGTCAACTCAATGCATATTATGTAGACT 2384
LBClus4  GGGAGATGATATTTGTGAAGGCAGTACGCATTGGCCAGCTCAATGCATACTATGTAGACT 2384
         ** **   *   *    * *   *  * *****  *   *****   ** ******   *
Possible frame-shift site  Possible start of free LBC-ori and LBClus4 RdRp  Stem-loop (ΔG=-17.9 kJ/mol, ΔG=-16.1 kJ/mol)
LBCbarr2 TGAAGTTTTGTGATGCAGCATCAAAAGAAATTACACGTCAAGCAGGAGCAACGATGTGTG 2406
LBC-ori  ATAAGAACGTCACAAATTATAGTCTTAAAACCGCTGCTCAAGTAGGAGCGACGATATCAA 2444
LBClus4  ATAAGAACGTCACGAATTACAGTCTTAAAACTGCTGCTCAAGTGGGAGCGACGATATCAA 2444
           ***                     ***   *   *****  ***** ***** *

LBCbarr2 GTAACTTCAGACACTTGTTTGCGAACTCAACTGGTACAGTATACGATTGGCTGGACCGGG 2466
LBC-ori  ATAACTTACGCCACGGATTTGTTGACAATCAACAAGACGCATACACGCGCTTGGTTGCCA 2504
LBClus4  ACAACTTACGTCATGGATTCGTTGACAACCAGCAAGACGCATACACGCGCTTGGTTGCCA 2504
           ***** * **    ** *   **            * ****    *  ***

LBCbarr2 ACAAAACCGTATTAAGAATGTACGACAGGCTTGCTAGTTACAAGTATGACTACAATATAG 2526
```

```
LBC-ori   ACTACTCTG---ATACGCGGAAGTGGATACGTGACAATTTTACATATAATTATAAT---A 2558
LBClus4   ATTACTATG---ATACACGGAAGTGGATACGTGACAATTTTACATATAATTATAAT---A 2558
          *  *    *     *    * *    *  * **  * **  *  *** * ** ***

LBCbarr2  CTGATAAAATAACAGAAAAAATAAGTCAGTTTCATCATGTACACGTGAGGCTGAAGGACT 2586
LBC-ori   TGGAGAAAGAAAAGTATAGGATAACCCAATACCACCATACACATGTGAGGTTGAAAGATT 2618
LBClus4   TGGAGAAAGAAAAATATAAGATAACTCAATATCACCACACACACGTTAGATTGAAAGATT 2618
            ** ***  **   * *  ****  ** *  ** **   *** ** **  **** ** *

LBCbarr2  TATATGCTAATAAGGATATTGTGACAGTAAACGGTCATGAAGCATATCTCGTGAACTTAC 2646
LBC-ori   TGTTTCCATCCAGGAAAATAGTTAAACTAGAGGGATATGAAGCCTTGTTGGCAATGATGC 2678
LBClus4   TGTTTCCATCTAGGAAAGTAGTCAAACTAACAGGATACGAGGCCCTACTGGCAATGATGT 2678
          * * * *    * * *  * ** * * **   **  * ** **     * *  *   *
               AGGTTTAACACTATAGAGTCAACGCATGTTACTTTCTTCACATATTTAAGAGCAC
LBCbarr2  TTGATAAGTACACAGACAAAGCTATTACACATGCTACTTTCATAACCTATCTAAGAGCAC 2706
LBC-ori   TAGACAGGTTTAACAACATAGAGTCAACACATGTAACTTTCTTCACATATTTAAGAGCAC 2738
LBClus4   TAGATAGGTTCAACAATATAGAGTCAACACATGTCACCTTTTTTACATACTTGAGGGCAT 2738
          * ** * **  *   * * **     *******  ** **  * ** **  * ** ***
                  5’ extra ScV-M1-EX231 (94%)       ATTAAGTATGGTAAAATACACC
          TACCTGATCGTGAAAAATAAA                                ATGGAAC
LBCbarr2  TGCCTGTTAGGGAAGCAAGGCTCTTCATAAAATTAGTCATTAAGTATGGTAGCATAGACC 2766
LBC-ori   TACCTGACCGTGAAAAAGAAGTCTTTATTAGCTTAGTCTTAAACTATAATGGCCTTGGCA 2798
LBClus4   TGCCTGATCAAGAGAGGGAAATCTTCATTAGCTTAATTTTGAACTACAACGGGCTTGGTA 2798
          * ****     **        **** ** *  *** *  * ** **     *  * *
          AAGCATGG       Homo sapiens chromosome 18 (93%) / Vitis vinifera contig VV78X249912.5 (80%)
          AAGAAAGGTTAAAAGAATAAGCAA--GAAGGAAAGAAAATAAAGGGC
LBCbarr2  AAGAATGGTTAAAAGAAGAAGGACTGTAGCAAAGCAAATACAGGGCAGCACTGACTTTG 2826
LBC-ori   GAGAGTGGTTGAAGTCTGAAGGTGTTAGGGCTAAACAAGCACAAGGTACTGTGAAATACG 2858
LBClus4   GAGAGTGGTTAAAGTCAGAAGGTGTTAGGGCAAAGCAAGCGCAAGGTACTGTAAAGTATG 2858
           *** ***** **    *****   *   ** ** ***   ** ** *      * *  *

LBCbarr2  ATCTAAGCAAAATTTTTGAGCTTAATGTACTGCATAATCGGGTTGAAACTACAGTTGATT 2886
LBC-ori   ATATGAGTAAACTATTTGAACTGAATGTACTAGAGAACGGAGTTGACGAAGAAGTTGACT 2918
LBClus4   ATATGAGTAAATTATTTGAGCTCAATGTGTTGGAAAATAGGGTTGACGAAGAGGTTGACT 2918
          ** * ** *** * ***** ** *****  *  * **  * *****       ***** *

LBCbarr2  GGAATGAAGAAAGAGAACATAGGACGCATCCTGATACAGTGAATATATCTTATCCAGATG 2946
LBC-ori   GGGAGAAAGAGAAACGCAACAGGTCAGATATCAAGACTGTTAACATAAGTTATGCAAAAG 2978
LBClus4   GGGAAAAAGAAAAACGGAACAGGACGAACATCAAGACTGTAGACATAAGTTATGCAAAAG 2978
          ** *  **** * *    * *** *  *     * ** **  * ***  **** ** * *
                          AGAATTTTTTAAATTAGCAAAACAAGAAGG
LBCbarr2  TCTATACTAAGTGCAGAGAATTGTTCGAATTAGCAAAGCAGGAAGGAAAGTCACCTATTA 3006
LBC-ori   TTCTCGAACATTGTAGAGAGCTATTCATCATGGCGAGGGCCGAAGGGAAACGGCCAATGA 3038
LBClus4   TTCTTGAGCACTGTAGAGAACTATTTATCATGGCAAGGGCTGAAGGAAAGCGGCCAATGA 3038
          *        * ** *****  * **     * ** * *   ***** **    ** ** *
                                    Marinilactibacillus sp. (93%)
LBCbarr2  AGATGGACTGGAATAAGTACTGGATGCAGCGAGCCTCAATAATGCCATCTGGTGCAGTCC 3066
LBC-ori   GGATGAAATGGCAAGAGTACTGGAGGCAGAGAGCAGTTATCATGCCAGGTGGATCGGTCC 3098
LBClus4   GGATGAAATGGCAGGAGTATTGGAGGCAGAGAGCAGTTGTGATGCCAGGCGGTTCAGTCC 3098
           **** * *** *  **** **** **** ****     * ******   **  * ****

LBCbarr2  ACTCACAACATAGTGAGCTTGACAAGTATGTGAGGAAATTACCTCGTGAAGTCAAAAACA 3126
LBC-ori   ACAGTCAACATCCAGTCGAACAGGACGTGATTAGAGTATTACCCAGAGAAATCAGAAGTA 3158
LBClus4   ACAGTCAGCATCCAGCCGAACAAGATGTGATTAAGATATTACCCAGGGAAATTAAGAGTA 3158
          **   ** ***   *      *  *     * *    ******  * *** * *  *  *

LBCbarr2  AGAAGGGGTTGGCTGCCTCGCTACCTAATATAGACCAGAGGTACTTTCTGAGTAAAATAC 3186
LBC-ori   AGAAGGGGGTGGCAAGTGTCATGCCATACAAAGAACAGAAGTATTTCACGTCCAGAAGGC 3218
LBClus4   AGAAGGGGTTGGCAAGTGTTATGTCATATAAAGAACAAAAATATTTCACATCAAGGCGAC 3218
          ******** ****        *  *  * * *** ** *  ** **       *     *

LBCbarr2  CTTCTATTGAAGCGTATGTCAGTACAAAGTATGAATGGGGAAAAGTCAGGGCTTTATATG 3246
LBC-ori   CGGAAATACACGCTTACACTTCAACGAAATACGAGTGGGGAAAAGTGAGGGCACTATATG 3278
LBClus4   CAGAAATACATGCTTACACTTCCACAAAATATGAGTGGGGAAAAGTGAGAGCGTTGTACG 3278
          *    **  * ** **       ** ** ** ** *********** ** **  * ** *
                Highly conserved RdRp domain        TTATTAAATTT[A]TGAAGATACT
LBCbarr2  GATGTGATTTCAGCAGTCATGTTAATGCAGACTTTGGATTATTAAATTGTGAAGATACCT 3306
LBC-ori   GGTGTGATTTTTCATCACATACAATGGCTGATTTTGGATTGTTACAATGCGAGGATACAT 3338
LBClus4   GGTGTGACTTTTCATCACACACGATGGCTGATTTTGGGCTGTTACAGTGTGAGGATACAT 3338
          * ***** **       **    *  ** ** *****  * *** * ** ** ***** *
       Saccharomycopsis fibuligera chromosome B6 (89%)
          TTTCTTCACTATATA                  CTGAAGGGGGTCTCAAAACACTTTTGA
```

```
LBCbarr2 TCCCTCACTTTATACCTACTGGGCCACAAGCTACTGAGGGGTACGTAAAAACACTTTTAA 3366
LBC-ori  TCCCGGGCTTTGTACCAACAGGGTCTTACGCCAATGAGGATTATGTCAGGACCAGAATTG 3398
LBClus4  TCCCCGGATTTGTACCAACGGGGTCATATGCTAACGAGGACTATGTCAGGACTAGGATAG 3398
         ****    *** **** ** *** *  * ** *  ****  ** ** *  **     *
```

*Sus scrofa chromosome 7 (85%)*

```
         AAAGAGCTAAATA
LBCbarr2 AAACAGCTAAATATAATGTGCCTTTCTGTTATGACTATGATGACTTCAACAGTCAGCATT 3426
LBC-ori  CTGGGACTCACTCATTGATCCCTTTCTGTTACGATTTCGATGATTTCAACAGCCAACATT 3458
LBClus4  CAGGGACACACTCGTTGATTCCTTTCTGTTATGACTTTGATGATTTTAATAGTCAACACT 3458
               *  * *      * *********** ** *  ***** ** ** ** ** ** *

LBCbarr2 CAAAGTCAAGTATGCAAGCAGTGATAGACGCTTGGATAAATACATATTTCGATGACCTGA 3486
LBC-ori  CAAAGGAAGCCATGCAAGCAGTGATTGATGCATGGATATCTGTCTATCACGATAAGTTAA 3518
LBClus4  CAAAGGAAGCTATGCAAGCAGTTATCGATGCATGGATATCTGTTTACCACGATAAGTTAA 3518
         *****  *   *********** ** ** ** ******  *   **   **** *  * *

LBCbarr2 CTGAAGACCAGATTGCCTCAGCCCTATGGACTAGAGAAAGTGTTGGTGAAATGTTTGTTA 3546
LBC-ori  CAGATGACCAGATAGAGGCGGCAAAGTGGACACGAAACTCGGTAGATAGAATGGTCGCTC 3578
LBClus4  CAGATGATCAAGTAGAAGCAGCAAAGTGGACAAGAAACTCAGTAGATAGAATGATCGCAC 3578
         * ** ** **  * *   * **    *****  ** *    ** * *  **** * *

LBCbarr2 ATGATGTAAAGAACAGTGTCAGATATGAAGCCATGGGAACTCTGTTTAGCGGATGGAGGC 3606
LBC-ori  ACCAACCTAACACTGGTGAGACTTATGATGTTAAAGGGACACTGTTTAGTGGCTGGCGAT 3638
LBClus4  ACCAAACGAACACCGGCGAGACTTATGATGTCAAAGGGACACTATTTAGTGGCTGGCGAT 3638
         *  *    ** *   * *  *  ***** *  *  ** ** ** ***** ** *** *

LBCbarr2 TGACAACTTTTATAAACACCGCTCTTAATTATGCATATCTTGCTAAGGCAGGGATAAATA 3666
LBC-ori  TAACAACATTTTTCAATACGGCGTTGAACTATTGCTACCTGGCTAATGCAGGTATAAACT 3698
LBClus4  TAACTACATTTATCAACACAGCATTAAACTATTGCTACTTGGCCAATGCAGGTATAAACT 3698
         * ** ** *** * ** ** **  * ** ***   **  * ** ** ***** *****

LBCbarr2 AATTAACCAATATAAGTATCCATAATGGTGATGATGTCTATGCAGGGACGAGAAATTTGA 3726
LBC-ori  CACTAGTGCCAACGAGTCTCCATAATGGTGATGATGTTTTTGCAGGGATAAGGACAATAG 3758
LBClus4  CACTAGTACCAACGAGCCTCCACAACGGTGATGATGTCTTTGCAGGTATAAGGACGATAG 3758
          * **      *  **  **** ** *********** * ****** *  ** *   *

LBCbarr2 AAGATATTGCTAGGCTTTTGAAGAATAGCAAGCAGATGGGAATACGTGCCAATACCACGA 3786
LBC-ori  CTGACGGTATTTCTTTGATCAAAAACGCCGCAGCCACGGGAGTTCGCGCTAATACAACTA 3818
LBClus4  CTGACGGTATTTCATTAATCAGAAATGCGGCAGCTACTGGAGTACGTGCAAATACGATTA 3818
           **   *  *    *  * *  **          *  *** * ** ** ***** *  *

LBCbarr2 AAATGAGCATTGGTACTATCGCTGAGTTTTTACGTGTCGACATGAGGGCTGAAAAACCTA 3846
LBC-ori  AAATGAACATTGGTACGATAGCAGAGTTTTTGAGAGTTGATATGCGTGCAAAAAATAGTA 3878
LBClus4  AGATGAACATAGGAACAATAGCGGAATTTTTGAGAGTTGATATGCGTGCAAAAGACAGTA 3878
         * **** *** ** ** ** ** ** *****  * ** ** *** * **  ** *   **

LBCbarr2 CTAGTGCTCAGTACTTAACTAGAGGGATATCTACTTTCGTACATGGTAGGATAGAAAGTG 3906
LBC-ori  CTGGCAGTCAGTATTTAACAAGAGGGATTGCTACCTTCACGCACAGTAGGGTTGAGTCTG 3938
LBClus4  CTGGTAGCCAATATTTGACAAGAGGGGTTGCTACTTTTACACACAGTAGGGTCGAGTCAG 3938
         ** *    ** ** ** ** ****** *  **** **    **  ***** * **    *

LBCbarr2 AGGCTCCAGTCGGGTATAGGGCTATGGTTAGTGCTTATAAGACGCGATACGATGAAGTGG 3966
LBC-ori  ATGCACCACTGACATTGCGCAATCTAGTATCTGCTTACAAAACCAGATATGACGAGATTT 3998
LBClus4  ATGCCCCACTAACATTACGTAATCTAGTATCTGCATACAAGACCAGGTATGATGAGATCA 3998
         * ** *** *    *   *   * * **   *** ** ** **  * ** ** **  *

LBCbarr2 TAGAAAGAGGAGGAAATGCGCAACGACTAAGGCATTTGTATCGTAAACAACTATTTTTCG 4026
LBC-ori  TAGCTCGTGGCGCAAGCATCGATAACATGAAGCCACTCTATCGTAAGCAATTATTTTTTG 4058
LBClus4  TAGCTCGTGGTGCTAACGTTGATGATATGAAACCACTTTATCGGAAACAATTATTTTTCG 4058
         ***   * ** *  *      *     * *  *   * ***** ** *** ******* *

LBCbarr2 CTCGTAGAAAGTTTGATGTGTCTGAAGAAATGCAAGAAAAATTGTTAAAGACGCACGTGC 4086
LBC-ori  CTAGAAAGTTGTTCAATGTCGAGAAGGACATTGTTGACAATCTGATAACGATGGACATAT 4118
LBClus4  CTCGCAAGTTATTCAATGTCGAGAAGGGTATAATTGATAATTTGATAACGATGGACATAT 4118
         ** * *     **  ****     * *  **    ** **  ** *** ** * ** *

LBCbarr2 AAGCTGGCGGTCTATCAGTCAATGGTAAGATAGGAAACTACACATTGGAAGATGACAGCC 4146
LBC-ori  CATGTGGCGGTTTGCAAGAAAAGGGTAGGGTATCAGAGATGGTGTTACAGGAGGTTGACA 4178
LBClus4  CATGTGGTGGTTTGCAGGATAAAGGTAAGGTGTCAGAAGTGGTGTTACAGGAAGTTGATA 4178
          *  *** *** *    *  ** **** * *   * *       **  * ** *

LBCbarr2 TTGCATACGCAGATATTGACGTGAATGAAATAAGC------AGCTTATTACAGCCTGGAG 4200
LBC-ori  TTGAGAATATAGATAGTTATAGGAAGACAAGGATGATCGCCAAACTGATTGACAAGGGGG 4238
```

```
LBClus4   TTGAAAATATAGACAGTTATAAGAAGACTAGGATGATCGCAAAACTGATCGACAAGGGGG 4238
          ***   *   *** * * *   ***    *  *        *   *  *  *    ** *
           Stem-loop for packaging ΔG=-7.1 kJ/mol
LBCbarr2  TTAAGGACTACGTGACTAGTTTGAAGTCTTTGTATCCGGAAATACGGGAATTTATAACCG 4260
LBC-ori   TTGGCGATTATACTGCATTCCTGAAAACTAACTTTTCCGAGATAGCTGATGCTATCACAA 4298
LBClus4   TTGGTGATTACACAGCATTCTTAAAAACTAACTTCTCCGAAATAGCTGATGCTATCACAA 4298
          **   ** **     *     * **  **   *   * ** ***   **   *** **

LBCbarr2  AAAAGACAGTGAGAAGGACACTTATGAAAGCCTTCAACATAAATAAAAACACGATATGTA 4320
LBC-ori   GAGAGACACGTGTAGAGTCAGTGACCAAGGCTTATAATGTTAAGAAGAAAACGGTCGTAC 4358
LBClus4   AGAAACATGTTGTAGAGTCATTGACCAAGGCCTACAATGTCAAGAAGAAAACGGTCAGAA 4358
             *         *  * ** * *  ** ** *  **  * ** ** ** *** *

LBCbarr2  TCAAACCAGCCAGTAGCAAGGCGATATATAATCAGATAGCACTAAGAGGTGCATGGAATG 4380
LBC-ori   GCGCGTTTAGGGACCTAAGCGCAGCATATCATGAAAGAGCGGTGAGACATGCTTGGAAGG 4418
LBClus4   GCGCGTTTAGGGACCTAAGCGCAGCATATCATGAGAGAGCAGTTAGACATGCTTGGAAGA 4418
           *               *  **   **** ** * * ***  * ***  *** *****
         Stem-loop for packaging (ΔG=-52.3 kJ/mol)
LBCbarr2  ATGACTCTAATTTCAGAATATTTAACAGAGTTAGACAGGGTGTGAGTAACGTGATAGGCG 4440
LBC-ori   GGATGAGTGGACTACACATAGTCAACAGGATTCGTATGGGAGTGAGCAACTTAGTAATGG 4478
LBClus4   GGATGAGCGGACTACATATAGTTAACAGGGTTCGTATGGGCGTAAGCAACTTAGTAATGG 4478
                      *    *** * *****  ** *   *** ** ** *** *  **   *

LBCbarr2  TATTAGGTAAACTGTCCAATGCGCATGCAAAAGTATTAAGCGAAACAAATGACCCTATCA 4500
LBC-ori   TTGTTAGCAAAATCAATCCTGCAAAAGCTAATGTGCTAGCCAAATCAGGAGATCCTACAA 4538
LBClus4   TTGTTAGCAAAATCAATCCTGCGAAAGCTAATGTGTTAGCCAAATCAGGGGATCCCACCA 4538
          *  *  * *** *      ***  * ** ** **  **  * ** **   ** ** *  *
                                                    Stop of LBCbarr2 Gag-Pol
LBCbarr2  AATGGTTGTCTATATATATAACATAGATGTAATACCAAGAAAGTTGTGACCTATATGACA 4560
LBC-ori   AATGGCTTGCAGTCCTTACATGATATACAGGCAACCACATAAGACCTGAGAACAAAGAGT 4598
LBClus4   AATGGCTTGCTGTCCTTACATGATATACAGGCAACAACATAAGACCTGAGAACAAAGAGT 4598
          ***** *  *  *   ** *  *** *      ** *   ***   ***     * **
                         Stop of LBC-ori and LBClus4 Gag-Pol          Stem-loop for replication (ΔG=-13.8 kJ/mol)
LBCbarr2  ACGGC------------     4565
LBC-ori   ACATACGATACTACGCA     4615
LBClus4   ACATACGATACTACGCC     4615
          **
```

*Stem-loops for replication in LBV-ori (ΔG=-7.5 kJ/mol and ΔG=-2.9 kJ/mol, respectively)*

**Figure S1**. Multiple sequence alignment between ScV-LBC1-original, ScV-LBClus4, and TdV-LBCbarr2 (+) strand nucleotide sequences (cDNA). 5′GAA(A/T)TT conserved motif (5′ conserved), translation initiation (start of Gag and Gag-Pol, or internal possible start ATG in Pol ORF of LBC1-original, LBClus4 and LBCbarr2), termination codons (stop of Gag and stop of Gag-Pol), ribosome frameshifting site (-1 frameshift site), frameshifting associated sequence (stem loop for frameshift), packaging signal (stem loop for packaging), and replication signal (stem loop for replication) are indicated, shaded and/or underlined in the nucleotide sequence. The highly conserved RdRp domain located in the central third of Pol is also underlined. ∩, ribosomal frameshift. Asterisks (*) indicates identical nucleotide positions. Sequence stretches of V-LBC genomes sharing high local identity with some mitochondrial or genomic sequences of several organisms are brown shaded, the sequences of other organisms are shown above each homologous stretch of LBC genome, and each percentage of identity is in parenthesis.
